# A broad-spectrum antibiotic adjuvant SLAP-S25: one stone many birds

**DOI:** 10.15698/mic2020.08.726

**Published:** 2020-06-15

**Authors:** Meirong Song, Kui Zhu

**Affiliations:** 1 College of Veterinary Medicine, China Agricultural University, No.2 Yuanmingyuan West Road, Beijing 100193, China.

**Keywords:** antibiotic adjuvant, Gram-negative bacteria, LPS, phosphatidylglycerol

## Abstract

The rapid emergence of antibiotic resistance has caused serious threat to global health. The worldwide search for novel classes of antibiotics to combat multidrug-resistant (MDR) bacteria is barren since about half a century ago. One of the promising strategies to combat the MDR pathogens is the combinational therapy. For instance, trimethoprim and clavulanic acid are routinely used to enhance the efficacies of sulfonamides and β-lactam antibiotics in clinic, respectively. Nevertheless, such adjuvants are specific for certain classes of antibiotics. We hypothesized that the combinational treatments with antibiotic adjuvants targeting the bacterial membrane may potentiate other antibiotics against MDR Gram-negative pathogens. In our recent publication (Song *et al.*, doi: 10.1038/s41564-020-0723-z), we demonstrate a short linear antibacterial peptide SLAP-S25, which potentiates multiple antibiotics with different modes of action against Gram-negative bacteria. The mechanism studies show that SLAP-S25 targets both lipopolysaccharide (LPS) in the outer membrane and phosphatidylglycerol (PG) in the inner membrane of *Escherichia coli*. The impaired bacterial membrane caused by SLAP-S25 promotes the intracellular accumulation of antibiotics in bacteria. Our results indicate that the bacterial membranes are promising targets for the discovery of new antibiotics or antibiotic adjuvants to combat MDR bacteria associated infections.

The structure and functions of bacterial membranes are of vital importance to maintain bacterial homeostasis, especially for Gram-negative bacteria. The accessorial and impermeable outer membrane, consisting of a dominant component (LPS), protects bacteria from antibacterial insults.

## TARGETING LPS IS UNIQUE FOR GRAM-NEGATIVE PATHOGENS

The outer membrane is a crucial permeable barrier for Gram-negative bacteria to prevent harmful detergents and antibiotics from entering into bacterial cells. Most hydrophobic antibiotics like macrolides have less or no antibacterial activity against Gram-negative bacteria because of the inability to access the outer membrane. Therefore, antibiotics that permeate into or disrupt the outer membrane are one of the prerequisites against Gram-negative pathogens. For example, colistin, a last-resort antibiotic against Gram-negative bacteria in the clinic, targets the LPS. However, the rapid dissemination of the plasmid-borne genes *mcr* gives rise to colistin resistance, leading to few therapeutic options in the clinic. Our synthesized SLAP-S25 targets either wild-type LPS or phosphoethanolamine modified LPS (pEtN-LPS; mediated by the enzyme encoded by *mcr* genes) with similar minimum inhibitory concentrations (MICs) of SLAP-S25 against both *mcr-*positive and *mcr*-negative bacteria. Notably, SLAP-S25 has stronger abilities than colistin to increase the permeability of the outer membrane in *mcr*-positive *E. coli*. Targeting LPS is the first step for SLAP-S25 to potentiate the efficacy of antibiotics against Gram-negative bacteria.

## PG IN THE INNER MEMBRANE IS TARGETED BY SLAP-S25

The bacterial inner membrane is responsible to maintain crucial cellular events, such as nutrient intake, respiration, and division. The inner membrane is mainly formed by three types of phospholipids including zwitterionic phosphatidylethanolamine (PE), anionic PG and cardiolipin (CL) in both Gram-positive and Gram-negative bacteria. Targeting any components may disrupt the intrinsic structure and function of the inner membrane, resulting in bacterial death. We found that SLAP-S25 shows high affinity to PG, disrupting the integrity of the inner membrane and thereby increasing permeability. PG is the predominant component in bacteria, whereas there is trace amount of PG in mammalian cells except the surfactant of the lung. Consistently, SLAP-S25 displays no hemolysis and cytotoxicity to diverse mammalian cells. Up to now, only few antibiotics targeting PG have been reported and used in the clinic. The verification of PG as a promising antibacterial target opens a supplementary avenue to the screening of other compounds to circumvent antibiotic resistance. In addition, it is always difficult to develop antibiotic resistance through targeting a specific compound in the membrane due to high fitness cost in bacteria.

## COLLECTIVE BEHAVIOR OF SLAP-S25 LEAD TO THE INTRACELLULAR ACCUMULATION OF ANTIBIOTICS

**Figure 1:**
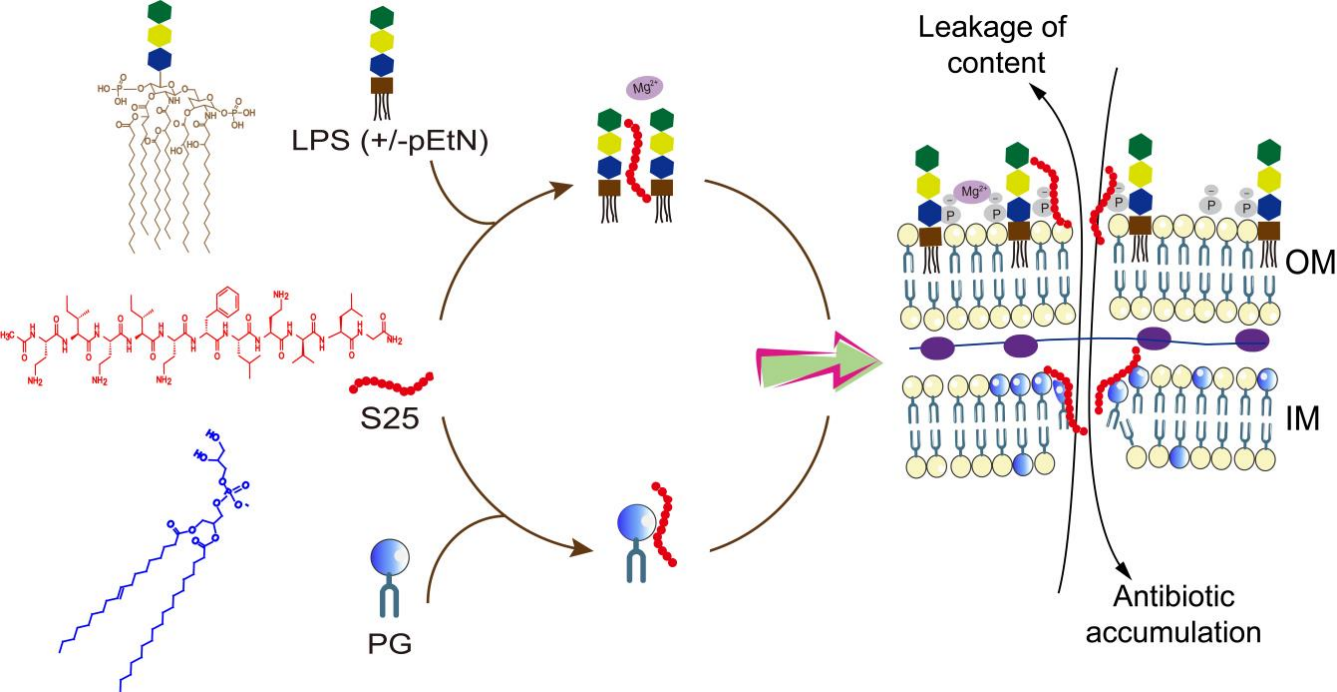
FIGURE 1: Mechanism of the synergy of SLAP-S25 in combination with antibiotics. SLAP-S25 targets both LPS and pEtN-LPS by competing with Mg^2+^ to dissect the tight joint between two neighboring lipids of LPS to increase the permeability of the outer membrane. pEtN-LPS is a modified LPS molecule. Importantly, SLAPS25 binds to the terminal group in the glycerol of PG, disrupting the integrity and function of the inner membrane. The collective behaviors of SLAP-S25 finally result in the intracellular accumulation of antibiotics to potentiate antibiotic efficacy.

The routine antibiotics commonly bind to specific targets, and bacteria quickly evolve resistance to each antibiotic using different strategies. The collective behavior of SLAP-S25 demonstrates that SLAP-S25 can recognize at least two targets (LPS and PG) to exert antibacterial activities (**Figure 1**). Targeting both LPS in the outer membrane and PG in the inner membrane achieves synergies between SLAP-S25 and multiple antibiotics. The damaged membrane in bacteria results in the accumulation of intracellular antibiotics to kill bacterial pathogens. In such a scenario, there is low probability for bacteria to simultaneously develop resistance or protection due to the increase of metabolic burden. Thus, the collective behavior of antibiotic candidates may endow them with an extended lifespan when introduced in the clinic. In addition, the combination of SLAP-S25 with antibiotics such as colistin, can not only enhances the efficacy of antibiotics but also reduce the amount of antibiotics to diminish their side effects and the selective stress for other bacteria, facilitating the containment of the prevalence of MDR Gram-negative pathogens.

## PERSPECTIVE

To counter the disturbance by antibiotics, bacteria always initiate defensive responses to maintain cellular hemostasis. We found the upregulation of genes related to membrane repair for either the outer membrane or the inner membrane in the presence of SLAP-S25, based on transcriptome analysis in *E. coli*. For example, the gene responsible for the synthesis of exopolysaccharide production protein yjbE and the genes encoding phage shock response proteins were obviously upregulated. Remarkably, the phage shock response proteins detect and mitigate various insults causing the permeability of bacterial inner membrane. Interestingly, these results are consistent with our observations that SLAP-S25 binds to either LPS or PG in *E. coli*. Nevertheless, it remains unclear how such cellular responses modulate the outcomes of SLAP-S25 treatment and the sequential signal transduction in bacteria. Our ongoing works will elucidate the underlying mechanism of SLAP-S25 against MDR Gram-bacteria, shedding light on the discovery and development of novel antibiotics or antibiotic adjuvants.

